# **Do pre-schoolers with high touchscreen use show executive function differences?**

**DOI:** 10.1016/j.chb.2022.107553

**Published:** 2023-02

**Authors:** Ana Maria Portugal, Alexandra Hendry, Tim J. Smith, Rachael Bedford

**Affiliations:** a Centre for Brain and Cognitive Development, Birkbeck, University of London, UK; b Center of Neurodevelopmental Disorders (KIND), Division of Neuropsychiatry, Department of Women's and Children's Health, Karolinska Institutet, Stockholm, Sweden; c Development and Neurodiversity Lab, Department of Psychology, Uppsala University, Sweden; d Department of Experimental Psychology, University of Oxford, UK; e Department of Psychology, Institute of Psychiatry, Psychology & Neuroscience, King's College London, UK; f Child and Adolescent Psychiatry Department, Institute of Psychiatry, Psychology & Neuroscience, King's College London, UK; g Department of Psychology, University of Bath, Bath, UK

**Keywords:** Executive function, Toddler, Mobile touchscreen media, Working memory, Cognitive flexibility, Inhibitory control

## Abstract

The recent increase in children's use of digital media, both TV and touchscreen devices (e.g., tablets and smartphones), has been associated with developmental differences in Executive Functions (EF). It has been hypothesised that early exposure to attention-commanding and contingent stimulation provided by touchscreens may increase reliance on bottom-up perceptual processes and limit the opportunity for practice of voluntary (i.e., top-down) attention leading to differences in EF. This study tests the concurrent and longitudinal associations between touchscreen use (high use, HU ≥ 15 min/day; low use, LU < 15 min/day), and two components of EF (working-memory/cognitive-flexibility, and impulse/self-control), building explicitly on recent developmental models that point to a bidimensional structure of EF during toddlerhood and pre-school years. A longitudinal sample of 46 3.5-year-olds (23 girls) was tested on a battery of lab-based measures and matched at 12 months on a range of background variables including temperament. Touchscreen HU showed significantly reduced performance in lab-based Working Memory/Cognitive Flexibility, although this became non-significant when controlling for background TV. Impulse/Self-control was not significantly associated with touchscreen use but was negatively associated with non-child-directed television. Our results provide partial support for the hypothesis that using touchscreen devices might reduce capacity for top-down behaviour control, and indicate that broader media environment may be implicated in early executive function development. However, it may also be the case that individuals who are predisposed towards exogenous stimulation are more drawn to screen use. Future studies are needed to replicate findings, demonstrate causality, and investigate bidirectionality.

## Highlights

• This study tested Executive Functions and touchscreen use in 3.5-year-olds.
• High touchscreen users had reduced Working Memory/Cognitive Flexibility.
• Impulse/Self-control seemed to be related to the broader family media environment.
• Future work should investigate causality and different types of platforms and use.

## 1 Introduction

Mobile touchscreen media (e.g., smartphones and tablets) are an integral part of family life. However, concerned parents, policymakers, and scientists have long questioned the potential impact on infant and toddler cognitive development, although rigorous research addressing the associations between touchscreen media and cognitive development in the early years is limited. It has been hypothesised that the daily exposure to fast-paced, attention-commanding stimulation provided by digital media devices may limit pre-schoolers’ opportunity for critical practice of voluntary (i.e., endogenous) attention and cognitive control – which encapsulate executive functions (EFs) (Zimmerman & Christakis, 2007). However, most studies to-date have not tested such longitudinal associations with touchscreen use whilst controlling for pre-existing individual demographic or precursor differences in EFs.

For television viewing, although the evidence is considered to be inconsistent (Kostyrka-Allchorne et al., 2017b) more exposure to various patterns of television viewing (child or adult-directed, household viewing) has often been associated with concurrent and/or long-term attention difficulties (Geist & Gibson, 2000; Kostyrka-Allchorne, Cooper, Gossmann, Barber, & Simpson, 2017; Zimmerman & Christakis, 2007) and EF problems (including inhibitory control, IC, working-memory, WM, and cognitive flexibility, CF (Barr, Lauricella, Zack, & Calvert, 2010; Corkin et al., 2021; Nathanson, Alade, Sharp, Rasmussen, & Christy, 2014). Thorough examination of the mechanisms for and the direction of the effects are limited, but evidence from experimental studies suggests an immediate, short-term negative impact on EFs (Kostyrka-Allchorne, Cooper, Kennett, Nestler, & Simpson, 2019; Lillard, Drell, Richey, Boguszewski, & Smith, 2015; Lillard, Li, & Boguszewski, 2015; Lillard & Peterson, 2011).

The media environment of preschool children involves a dynamic mixture of television, videogaming, tablets, and smartphones use. When considering this media amalgam, recent research shows that screen time (an aggregation measure of TV, computer, and touchscreen devices use) is linked negatively with EF performance (longitudinally from 2 to 3 years of age, McHarg, Ribner, Devine, & Hughes, 2020; and amongst 8- to 36-month-olds both prior to and during the Spring and Winter 2020COVID-19 pandemic lockdowns, (Hendry et al., 2022). Experts in the field have argued that modern touchscreen platforms (a fundamentally different platform than television) might be a more developmentally appropriate media due to its interactive nature (Christakis, 2014; Geist, 2014; Zimmermann, Moser, Lee, Gerhardstein, & Barr, 2017), which could better support learning through active exploration (Christakis, 2014), and personal and contingent responses (Kirkorian, 2018). Touchscreen devices have been hypothesised to influence EF via similar mechanisms to TV viewing, namely displacing other activities which associate with increased EF (such as turn-taking during parent/peer interactions or game playing), as well as increasing exposure to rapidly changing and fantastical content via the digital screen directly depleting EF resources (Kostyrka-Allchorne et al., 2017b, Lillard et al., 2015, Lillard et al., 2015, Lillard and Peterson, 2011). The amplification of low-level feature salience on interactive screens, together with the physical proximity of the handheld device may exacerbate such effects.

Several studies have tested the isolated impact of modern mobile touchscreen devices on the development of EFs. McNeill, Howard, Vella, and Cliff (2019) found a negative association between pre-schoolers active media use (>30 min/day of playing with apps on touchscreen-like platforms) and cool-IC measured one year later (indexed by performance on a Go/No-go task), but no associations with WM and CF. Similarly, Lawrence, Narayan, and Choe (2020) found a concurrent negative association between pre-schoolers’ use of mobile devices and age of first use with IC, although no associations were found for parent-reported effortful control, and no other EFs were measured. In contrast, a recent study in infants found no evidence for associations between touchscreen exposure and IC (either parent-reported or lab-based) at 10 months of age, and reported only a weak concurrent positive association with parent-assessed EF more generally, potentially driven by a CF scale (Lui, Hendry, Fiske, Dvergsdal, & Holmboe, 2021).

These few studies to-date point to a potential negative link between mobile touchscreen use and EF in pre-schoolers, but, critically, they have so far not been able to demonstrate a directional association because they did not test for pre-existing individual differences in demographic or EFs emergent abilities. Pre-existing related traits (e.g. temperament) may affect the degree to which children engage in media use, which strengthens familiarity and pleasure during the activity, and can lead to more exposure (Radesky, Silverstein, Zuckerman, & Christakis, 2014). It is crucial to embed research within a multi-method longitudinal study that enables understanding of developmental processes and underlying mechanisms.

### 1.1 The current study

In previous studies with the same sample (Portugal, Bedford, Cheung, Gliga, & Smith, 2021; Portugal, Bedford, Cheung, Mason, & Smith, 2021), we have employed a longitudinal design within a prospective study of toddlers with different levels of use of touchscreen devices with application of neurocognitive methodologies to study EFs and attention. Across multiple tasks, we have repeatedly shown a bias to bottom-up attention orienting (Portugal, Bedford, Cheung, Gliga, & Smith, 2021; Portugal, Bedford, Cheung, Mason, & Smith, 2021), which could potentially lead to the displacement of opportunities for endogenous attention control (Portugal, Bedford, Cheung, Mason, & Smith, 2021) . In the current study, we wanted to understand the extent to which these biases and associated difficulties were observed in naturalistic scenarios that demanded executive function. Therefore, we tested group-level differences in EF between children who have different levels of touchscreen use. Importantly, at 12 months of age these children *did not differ* on a range of background variables including parent-reported temperament (such as effortful control), an infant precursor of EF, suggesting any later associations between touchscreen use and EF may not be due to existing group differences.

The different sub-dimensions of EF are thought to develop differently throughout early childhood, and it is still not clear how EF is organised early in life. We believe it is important to dissociate performance in EF in its sub-constructs to better understand the effects of media use. Based on Hendry, Jones, and Charman (2016) conceptual framework and the recent evidence of 3–4 year 10.13039/501100000353EF being better modelled within a bidimensional structure of self-control and working-memory/cognitive flexibility (Scionti & Marzocchi, 2021), and partly supported by the pattern of associations within our dataset, we created two composite measures of 10.13039/501100000353EF, one for working-memory/cognitive flexibility (which comprises both a cognitive flexibility measure and two working-memory measures) and another for impulse/self-control (which comprises both cool and hot inhibitory control).

If touchscreen use disrupts EF performance in pre-schoolers, as it has been shown before with TV and touchscreen media devices (Barr et al., 2010, Kostyrka-Allchorne et al., 2017b, Lawrence et al., 2020, Lillard et al., 2015, Lui et al., 2021, McNeill et al., 2019) then we predict differences in all components of EF in children who have high daily touchscreen use compared to matched children with low daily touchscreen use, such that children with high daily touchscreen use will demonstrate worse EF performance compared to children with low daily touchscreen use.

## 2 Methods

### 2.1 Participants

Fifty-six infants were recruited between October 2015 and March 2016, through the Birkbeck and Goldsmith’s Babylab databases and communication and social media. Three participants were later excluded from the study – one withdrew consent after the first visit, and the other two received a later diagnosis of genetic or neurological conditions. Families visited the Babylab as part of three longitudinal visits at 12 months (N = 53, 23 girls, M = 376 days, SD = 20), 18 months (N = 49, 22 girls, M = 540 days, SD = 21) and 3.5 years (N = 46, 23 girls, M = 1256, SD = 16). Full sample details and details on the full set of experimental measures conducted during the visits are reported elsewhere (Portugal, Bedford, Cheung, Mason, & Smith, 2021). One child was born prematurely at 32 weeks, and one child occasionally suffers from Reflex Anoxic Seizures – as both were able to fully perform the tasks their data were retained in the analysis. The study was approved by the author institution's ethics board and parents provided written informed consent at each visit.

The current analysis is focused on the 3.5-year visit when EF measures were collected. The forty-six (who contributed with data for the current study) sample characteristics for age and background measures are presented in Table 1.

**Table 1:** Descriptive and frequency statistics for high and low touchscreen media users at 3.5 years. Continuous data is presented as mean (standard deviation). Categorical data is presented as n (%). User groups differed in background TV: high users’ parents reported more background TV.

	Sample	Low users (<15 min/day)	High users (≥15 min/day)	Between-groups comparison
N	**46**	**19**	**27**	
Touchscreen use (min/day)	**38 (63)**	**3 (4)**	**62 (73)**	***p* < 0.001**
Sex (n Girls)	23 (50%)	12 (63%)	11 (41%)	n.s. (0.134)
Mother's Education				
School leaving or college	3 (7%)	0 (0%)	3 (11%)	n.s. (0.125)
University or postgrad	42 (91%)	19 (100%)	23 (85%)	
Missing, N/A	1 (2%)	0 (0%)	1 (4%)	
Age (days)	1256 (16)	1257 (14)	1256 (18)	n.s. (0.781)
Background TV (min/day)∗	**169 (140)**	**112 (147)**	**208 (123)**	***p* = 0.022**
MSEL Visual Reception t-score	67 (9)	64 (9)	70 (8)	n.s. (0.054)
More than one language at home	21 (46%)	7 (37%)	14 (52%)	n.s. (0.259)
English as first language at home	40 (87%)	17 (89%)	23 (85%)	n.s. (0.915)
Touchscreen use (min/day) at 12 months	21 (49)	14 (29)	26 (59)	n.s. (0.420)
Background TV (min/day)∗ at 12 months	174 (161)	116 (156)	214 (154)	***p* = 0.039**
MSEL Standard Score at 12 months	109 (11)	109 (12)	109 (11)	n.s. (0.845)
IBQ/ECBQ at 12 months				
Surgency	5.1 (0.6)	5.1 (0.5)	5.1 (0.7)	n.s. (0.939)
Negative Affect	3.3 (1.3)	3.0 (1.2)	3.5 (1.3)	n.s. (0.168)
Effortful Control	4.7 (0.9)	4.7 (1.0)	4.7 (0.9)	n.s. (0.859)

MSEL = Mullen Scales of Early Learning; IBQ = Infant Behaviour Questionnaire; ECBQ = Early Childhood Behaviour Questionnaire.

∗ Values that exceeded 3 standard deviations from the mean were trimmed (i.e. changed to be one more than the non-trimmed highest value).

### 2.2 Touchscreen media use assessment and study design

Parents reported on their infants' media use via an online questionnaire before coming to the Babylab, at 12 months, 18 months, and 3.5 years. Parents were asked about the duration of their child's use in hours and minutes: ‘On a typical day, how long does your child spend using a touchscreen device (tablet, smartphone or touchscreen laptop)?’ (Bedford et al., 2016). In addition, at 3.5 years, parents recorded in two 24-h online diaries the times the child was watching television (adult-directed; or child-directed), or using a touchscreen (at home before the visit). The parent-report measure of touchscreen use duration was significantly associated with the amount (hours/day) reported on these diaries (r_s_ (44) = 0.62, *p* < 0.001).

Following our pre-registered analysis plan (https://doi.org/10.17605/OSF.IO/FXU7Y), the sample was divided into two groups based on the median of the parent-report daily touchscreen time at 3.5 years. Based on the median split, high users (HUs) had ≥15 min/day, and low users (LU) < 15 min/day. This dichotomous group variable was the main independent variable of interest and was chosen very early in the study design (it is a common approach when studying naturalistic media use with modest sample sizes; Barr et al., 2010; McNeill et al., 2019). Parent-report measures of screen time are known to be biased (Lin et al., 2015; Orben & Przybylski, 2019), but the rank order of individual differences would be expected to remain similar (Lin et al., 2015). We chose to use a binary variable to minimise the impact of such reporter bias. However, we have also included exploratory bivariate correlations using continuous touchscreen use. The groups matched on a range of general demographic covariates: sex, mother's education (below degree; or degree level or above), bilingualism (one language at home, or more than one language at home), English as the first language at home (yes, or no), age at 3.5 years, as well as general development level [assessed by the Mullen Scales of Early Learning (Mullen, 1995)] at 12 months (full scale) and 3.5 years (visual reception scale). Further, the groups matched in parent-reported temperament (including effortful control, an EF precursor) at 12 months. See Table 1 for descriptive statistics and tests between concurrent usage groups.

The child's media environment is dynamic, and likely to vary depending on age, level of development and family characteristics [e.g. change of routines such as starting attending nursery or parents going back to work (Valkenburg & Peter, 2013)]. To investigate whether the associations between touchscreen media use and EF were specific to recent naturalistic use, we tested in follow-up models the prolonged use (i.e., longitudinal stable touchscreen use, coded based on the three longitudinal visits and described elsewhere (Portugal, Bedford, Cheung, Mason, & Smith, 2021) and the past use (12-month use, coded dichotomously as greater than or equal to 10 min/day).

### 2.3 Background TV viewing

Background TV viewing, in minutes per day, was assessed through parent-report on the online media questionnaire by asking the question: ‘On a typical day, how long is a TV switched on in your home?‘). It was not matched between groups, with parents of high users reporting higher total household television at 12 months and at 3.5 years. For this reason the effect of background TV was tested in follow-up analysis. The 24-h media diaries were used to compute duration (hours/day) of television viewing that was adult-directed or child-directed.

### 2.4 Stimuli and procedure: EF measures

The behavioural tasks used in this study were part of a 3-h protocol described elsewhere (Portugal, Bedford, Cheung, Mason, & Smith, 2021). The EF battery (∼30 min) was completed in a fixed order (first the touchscreen-based tasks, i.e. Delayed Alternation, and Go/No-go; followed by the table-top tasks, i.e. Spin the pots, Snack Delay, Dimensional Change Card Sorting, and Glitter Wand). Performance during the EF battery was video recorded. Research assistants (blind to group status) recorded online observations of the child's performance on all tasks, and later watched videos to code measures and screen for invalid trials.

### 2.5 Touchscreen-based tasks

The touchscreen-based tasks were presented using E-Prime 2.0 Professional (Psychology Software Tools, Sharpsburg, PA) on a desktop touchscreen monitor (15-inch LCD Elo Entuitive).

#### 2.5.1 Delayed alternation, indexing WM

**Fig. 1:**
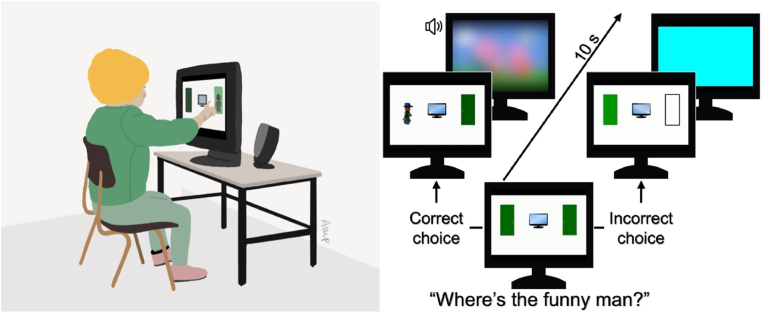
Touchscreen-based Delayed Alternation Task: on the left, illustration of a child doing the task; on the right, scheme of the task (the correct response was a tap on the door on the left-hand side of the screen).

A classical A not B task requiring the child to alternate responses (i.e., tap a door on the screen) between left and right of the screen on each correct successive trial, to watch a short entertaining video (*Peppa Pig*) – see Fig. 1. If the incorrect door was chosen, a boring sequence was displayed (a blue screen). The video/sequence duration (7 s) served both as a reward (if correct) and as a distractor (the child's eyes were drawn to the centre of the screen so that they would not use gaze to maintain a representation of which door to tap). The experiment included 4 demonstration and 19 testing trials. The duration of the sequence and number of trials were implemented after (Hendry, 2018) and other studies with this task (Wiebe, Espy, & Charak, 2008). The score was computed as the number of consecutive correct trials in the longest run of alternations minus the number of trials in the longest perseverative run (possible range −11 to 19), with a higher value representing a better maintenance of task demands (maintenance and updating) over the course of task.

#### 2.5.2 Go/no-go (“splat the cat!“), indexing cool-IC

**Fig. 2:**
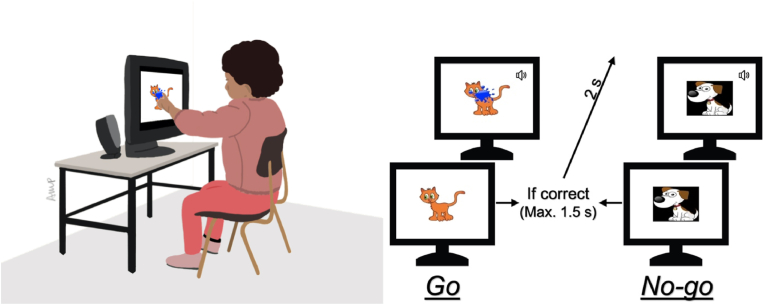
Touchscreen-based Go/No-go Task: on the left, illustration of a child doing the task; on the right, scheme of the task (a correct response in Go-trials is a tap within 1.5 s; a correct response in No-go-trials is no tapping within 1.5 s. If incorrect, a blank screen would be presented for 2 s).

A classic Go/No-go task requiring the child to tap the screen when a *more-frequent-cat* stimuli appeared (“*splat the cat”*, *Go* trial) but not when a *less-frequent-dog* appeared (“*don't splat the dog*”, *No-go* trial) – see Fig. 2. This version of the task has been previously used with children as young as 3 years (Hendry, 2018). The experiment included 8 demonstration and practice trials (fixed order), and 24 testing trials (randomized order with 75% *Go* trials and 25% *No-go* trials; *Go* trials were more frequent to elicite a prepotent motor response). In both *Go* and *No-go* trials, the stimulus was presented for 1.5 s or until the screen was tapped. Eight children had to be excluded from analyses because they refused to continue the task after the practice period (n = 3) or there were no online testing notes or videos available to screen for responses not registered by the touchscreen (trials where the child splatted the screen but the response was not registered by the touchscreen monitor; n = 5). The score was computed as the d prime (d′) sensitivity index, a standardized difference between the hit rate (proportion of *Go* trials to which there was a correct tap) and the false alarm rate (proportion of *No-go* trials to which there was an incorrect tap), calculated by subtracting the z-transform of the false alarm from the z-transform of the hit rate. A higher value represents a more efficient performance.

### 2.6 Table-top tasks

#### 2.6.1 Spin the pots, indexing WM

**Fig. 3:**
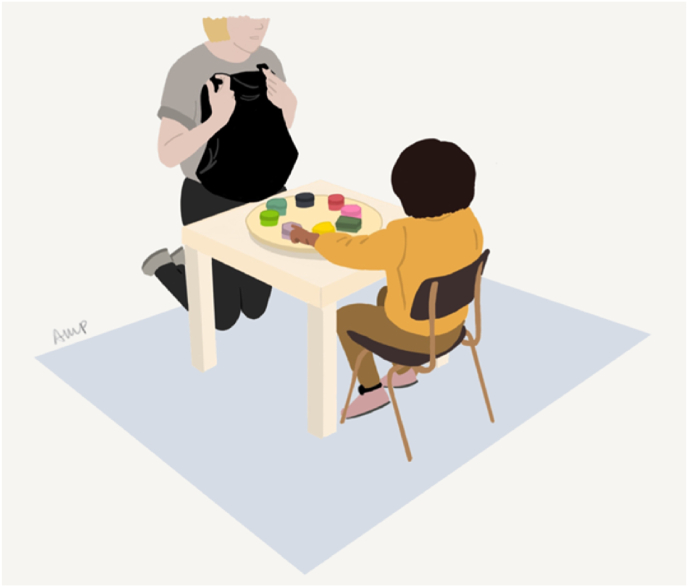
Illustration of a child doing the Spin the Pots task.

A multi-location search task resembling a “treasure hunt” game which followed the procedure previously described in (Hughes & Ensor, 2005). Eight visually distinct boxes were placed on a rotating tray and, while the child was watching, one craft gem was hidden in each of 6 boxes – see Fig. 3. The tray was covered, rotated, and uncovered again, and the child then encouraged to search for a gem by picking and opening a pot. The chosen pot was then emptied (if a gem was found) and put back onto the tray. This was repeated until all treasures were found, or for a maximum of 16 trials. The score was 16 minus the number of unsuccessful trials (possible range between 0 and 16). The most successful performance (=16) was achieved if all treasures were found in as few trials as possible (while maintaining and updating which pots were opened over the course of task).

#### 2.6.2 Snack delay, indexing hot-IC

**Fig. 4:**
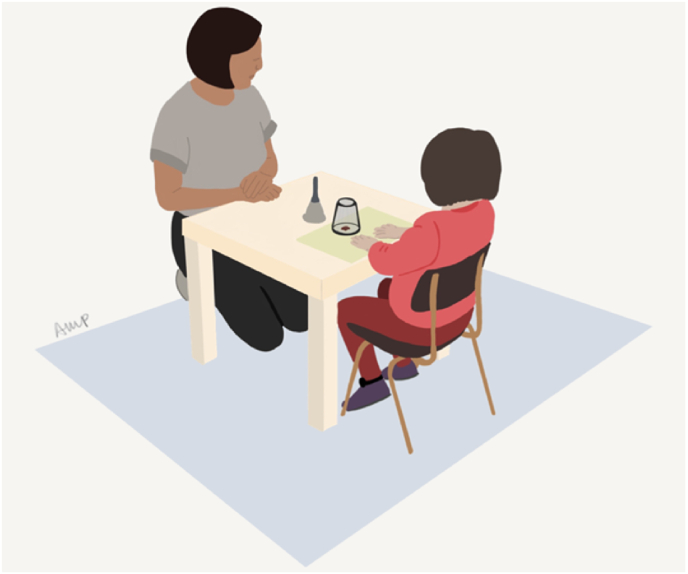
Illustration of a child doing the Snack delay task.

A delayed gratification task, adapted from (Kochanska et al., 1996) , introduced to the child as an occasion to get a treat (a paw-shaped dried fruit). A glass cup and a bell were placed on a placemat decorated with two handprints. After one treat was placed under the cup the child was instructed to wait still with their hands on the mat until the experimenter rang the bell – see Fig. 4. Four trials were done with delays of 10 s, 20 s, 30 s, and 240 s. Halfway through the delay, the experimenter lifted and lowered the bell without ringing it. During the last trial, where three treats were placed under the cup, the experimenter initiated a series of distractions (i.e., coughing, writing) and after lifting and lowering the bell, left the room for a period of 90 s, resuming the task when came back.

Performance was videocoded as a trial score ranging from 0 to 6 (0 = child ate the treat before the bell was lifted, 1 = ate the treat after the bell was lifted, 2 = touched the bell or cup before the bell was lifted, 3 = touched the bell or cup after the bell was lifted, 4 = removed both hands from the mat before the bell was lifted, 5 = removed both hands from the mat after the bell was lifted, and 6 = waited for the bell to ring). The score was a sum score of all trials (possible range 0–24, higher score indexing higher ability to delay gratification). Inter-rater reliability between the video coding was 0.992 (95% CI: 0.969–0.998, *n* = 10) assessed using a single measures 2-way mixed model. Data from this task has been previously reported in terms of associations with perfomance on a problem-solving task (Hendry, Agyapong, et al., 2022) but not in regards to touchscreen media use or any of the tasks reported in this study.

#### 2.6.3 Dimensional Change Card Sorting (DCCS), indexing CF

**Fig. 5:**
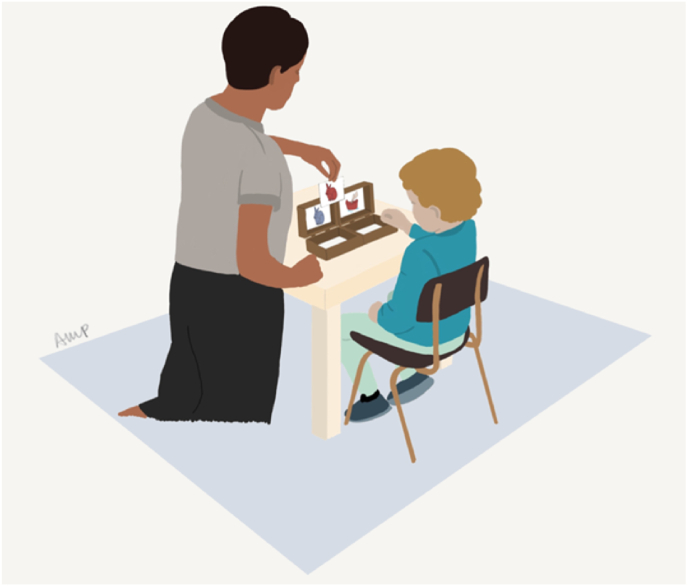
Illustration of a child doing the DCCS task.

The standard version of the Dimensional Change Card Sorting (DCCS, Zelazo, 2006), where children were required to sort a series of bivalent test cards into two sorting trays, first according to one dimension (e.g. color, pre-switch phase with 6 trials), and then according to the other (shape, post-switch phase with 6 trials) – see Fig. 5. There was a demonstration and a practice trial. On every trial the experimenter showed a card to the child, labelled it by the relevant dimension, and asked the child to sort it. Whether or not the child sorted correctly the experimenter simply proceeded to the next trial without reinforcing or correcting responses.

All children sorted correctly all pre-switch trials. The score was the number of cards successfully sorted on the post-switch phase.

#### 2.6.4 Glitter wand, indexing hot-IC

**Fig. 6:**
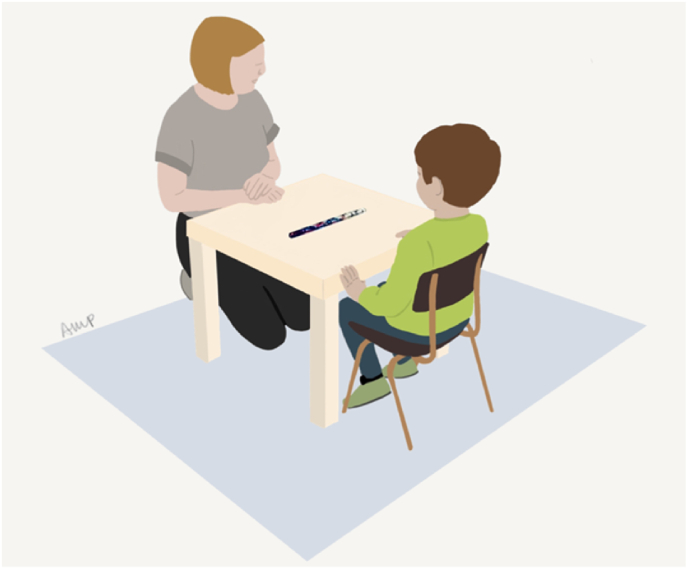
Illustration of a child doing the Glitter Wand task.

A Prohibition Task similar to the one described in (Friedman, Miyake, Robinson, & Hewitt, 2011). The experimenter drew the child's attention to an attractive toy (a glitter wand), placed it on the table within reach, and instructed the child to wait to touch the toy – see Fig. 6. The delay went for 30 s, when the experimenter verbally released the prohibition. Score was the latency to touch the wand (35 s if the child touched the wand only after release of prohibition, to distinguish cases where children touched the wand just before the release, in which case they were given score of 30 s).

### 2.7 Analytical approach

The 3.5-year visit analysis plan was pre-registered prior to data processing (https://doi.org/10.17605/OSF.IO/FXU7Y). Touchscreen group differences in EF measures at 3.5 years were proposed to be tested separately for screen-based and real-world tasks, using a multivariate analysis of variance (MANOVA) with each task index as dependent variables, and usage group as fixed factor. However, a deviation of this plan was necessary, as the associations between performance in the tasks in our dataset (as revealed by the correlations shown in Table 2) suggested a different organizational structure of EF, which is more in line with recent literature on the same age (Hendry et al., 2016; Scionti & Marzocchi, 2021). To fulfil the pre-registration intentions, a MANOVA with touchscreen usage group as a factor was reported in Appendix 1 of supplementary information. Additionally, univariate comparisons were also reported there for each task score.

**Table 2:** Correlations between observed measures. All scores were non-normally distributed (Shapiro-Wilk test p-value < 0.01) so correlations are represented as Spearman's rho rs (p-value).

Measure (Max N)	2.	3.	4.	5.	6. Glitter Wand Latency
1. Delayed Alternation Score (46)	0.337*(0.02)	**0.28’ (0.06)**	0.16 (0.35)	0.10 (0.52)	0.06 (0.72)
2. Spin the pots Score (46)		0.24 (0.11)	0.07 (0.66)	−0.15 (0.32)	−0.05 (0.76)
3. DCCS Correct sorted (46)			0.04 (0.84)	−0.16 (0.29)	−0.11 (0.48)
4. Go/No-Go D’ (38)				0.14 (0.41)	0.18 (0.27)
5. Snack Delay Score (46)					**0.396^∗∗^ (0.01)**

** p-value < 0.01, * p-value < 0.05, ‘p-value < 0.1.

Based on recent literature regarding the structure of EF in toddlers and pre-schoolers (Hendry et al., 2016; Scionti & Marzocchi, 2021) and on the correlations in Table 2, EF performance was dissociated into two different sub-components: Working-memory/Cognitive Flexibility and Impulse/Self-control. The Working-memory/Cognitive Flexibility component included the touchscreen-based Delayed Alternation task, the Spin the pots task, and the DCCS task. The Impulse/Self-control component included the touchscreen-based Go/No-Go task, the Snack Delay task, and the Glitter Wand task. Given the weaker associations between Go/No-go and the other two tasks, the analysis of this component was followed-up by dissociating hot (Snack delay and Glitter Wand) and cool (Go/No-go) IC.

The composite scores were computed as a mean value of all measures included in the score (task scores were scaled so they were 0–1, 1 being best performance). If a measure was missing the mean was calculated based on the available data points. The Impulse/Self-control composite was transformed to achieve a normal distribution (using an *lnskew0* transformation in Stata; StataCorp, 2013), which normalised the score; Shapiro-Wilk test *p*-value = 0.420).

The hypotheses on the pre-registration plan were non-directional, but because new evidence has been published since then (see introduction; Lawrence et al., 2020; McNeill et al., 2019) the predictions changed accordingly. It was predicted that high users of touchscreens would show poorer performance on the EF measures. To test for differences between high and low users in the EF outcomes, a MANOVA and separate univariate analysis of variance (ANOVA) were run.

## 3 Results

### 3.1 Association between touchscreen use and EF

Children's performance on the set of EF lab-based measures are presented in Table 3.

**Table 3:** Performance on EF lab-based measures at 3.5 years.

Continuous Measures	N	Mean	SD	Skewness	Raw range	% Ceiling
Working-memory/Cognitive Flexibility						
Delayed Alternation WM Score	46	.41	.19	.93	-4–19	2%
Spin the pots Score	46	.63	.30	-.76	5–16	13%
DCCS # Cards sorted	46	.43	.48	.32	0–6	39%
Impulse/Self-control						
Go/No-Go D′	38	.65	.28	-.84	0.59–3.30	13%
Snack Delay Sum Score	46	.81	.20	-1.90	10–24	24%
Glitter Wand Latency	46	.92	.23	-3.12	1–35	85%

A MANOVA model was run with touchscreen user group (HU versus LU) as a predictive factor of EF (Working-memory/Cognitive Flexibility and Impulse/Self-Control). The overall model was not significant, F (2, 43) = 2.269, *p* = 0.116, η^2^ = 0.095. However, the univariate between-subjects ANOVA on Working-memory/Cognitive Flexibility was significant, F (1, 44) = 4.498, *p* = 0.040, η^2^ = 0.093, with low users scoring higher than high users, see Table 4. Impulse/Self-Control did not significantly differ between groups, F (1, 44) = 0.074, *p* = 0.787, η^2^ = 0.002; neither did Cool Impulse/Self-Control (Go/No-go D’; U = 170, z = −0.178, *p* = 0.859), or Hot Impulse/Self-Control (mean between Snack Delay Score and Glitter Wand Latency; U = 260.5, z = 0.090, *p* = 0.928).

**Table 4:** Descriptive of the EF composite measures by touchscreen use group at 3.5 years.

	SAMPLE	LOW USERS	HIGH USERS
WORKING-MEMORY/COGNITIVE FLEXIBILITY			
MEAN (SD), N	0.49 (0.24) 46	0.57 (0.24) 19	0.43 (0.22) 27
IMPULSE/SELF-CONTROL			
MEAN (SD), N	1.60 (0.65) 46	1.56 (0.67) 19	1.62 (0.65) 27
*COOL* IMPULSE/SELF-CONTROL			
MEAN (SD), N	0.65 (0.28) 38	0.67 (0.26) 16	0.63 (0.29) 22
*HOT* IMPULSE/SELF-CONTROL			
MEAN (SD), N	0.87 (0.19) 46	0.85 (0.23) 19	0.88 (0.16) 27

Working-Memory/Cognitive Flexibility – Delayed Alternation, Spin the pots, DCCS.

To test for continuous effects, exploratory bivariate correlations were also run. Based on a box plot of 3.5-year touchscreen use, two outliers were removed (i.e. touchscreen usage 1.5 times the interquartile range above the upper quartile). Results were substantively similar to those reported in the main pre-registered analysis: the correlation between touchscreen use and IC (r = 0.052, *p* = 0.738) remained non-significant, while the association with WM/CF was in the same direction but no longer reached significance (r = −0.279, *p* = 0.067); p-values uncorrected for multiple comparisons.

### 3.2 Controlling for background TV

A follow-up MANCOVA model was run adding parent-reported background TV as covariate. Again, touchscreen user group was not a significant factor in the overall model, F (2, 41) = 1.678, *p* = 0.199, η^2^ = 0.076, neither was background TV, F (2, 41) = 1.534, *p* = 0.228, η^2^ = 0.070. In terms of Working-memory/Cognitive Flexibility, background TV did not have a significant effect, F (1, 42) = 0.270, *p* = 0.606, η^2^ = 0.006, but touchscreen group was no longer a significant predictor, F (1, 42) = 2.407, *p* = 0.128, η^2^ = 0.054. For Impulse/Self-Control, touchscreen group remained not significant, F (1, 42) = 1.048, *p* = 0.312, η^2^ = 0.024, but background TV was marginally significant, F (1, 42) = 2.863, *p* = 0.098, η^2^ = 0.064, with more background TV in the home being associated with poorer performance.

### 3.3 Associations between impulse/self-control and child/adult directed TV

To follow-up on this marginal effect of background TV on Impulse/Self-Control post hoc analyses were run using more detailed measures of child's TV viewing, based on parent media diaries. Cool Impulse Control (Go/No-go D′) was related significantly to viewing of adult-directed TV content (r_s_ (37) = −0.34, *p* = 0.039), but not with viewing of child-directed TV content (r_s_ (37) = −0.12, *p* = 0.495). Hot Impulse/Self-Control (mean between Snack Delay Score and Glitter Wand Latency) was not associated significantly with TV measures.

### 3.4 Longitudinal associations

There were no effects of touchscreen use at 12 months (past use) on lab-based EF at 3.5 years. There were also no effects of stable touchscreen use, i.e., longitudinal (from 12–18 months to 3.5 years) on EF performance. See results in Appendix 2 of supplementary information.

## 4 Discussion

This paper aimed to test the association between parent-reported touchscreen use and concurrent, objective, behaviourally-assessed executive function abilities in preschool children. Our study makes use of a longitudinal sample, matched at 12 months on a set of background demographics and temperament (precursors of EFs), to establish the temporal ordering of effects. Results showed that 3.5-year-old children who had a higher level of touchscreen use showed poorer performance on a composite experimental measure of CF and WM, although these effects became non-significant after controlling for background TV. Our findings are broadly consistent with previous reports of EF difficulties related to television viewing (Barr et al., 2010; Kostyrka-Allchorne et al., 2017a; Lillard, Drell, et al., 2015; Lillard, Li, & Boguszewski, 2015; Lillard & Peterson, 2011) and media use (Hendry et al., 2022, McHarg et al., 2020); but contrast with a recent report of positive concurrent associations of touchscreen exposure and EFs at a much younger age (10 months), after controlling for sociodemographic variables (Lui et al., 2021). Further, two recent studies, across a similar age period to our study, showed associations between mobile devices media use and IC, but no associations with WM or CF (Lawrence et al., 2020; McNeill et al., 2019), while in our study we found no associations with a composite measure of impulse/self-control.

What could explain the association between high touchscreen use and reduced CF and WM? During the fourth year of life there is a transitional shift from perseverative behaviour (necessary to learn regularities or rules in the world) to flexible behaviour (necessary for the generation of alternative behaviours in new situations), which is often tapped by the DCCS and partially by WM measures (Munakata, Snyder, & Chatham, 2012). Problems with the DCCS have been explained by a poorer ability to outcompete (i.e., not persevere) latent stimuli-response representations formed during the pre-switch DCCS phase (i.e., the learned rule), which is linked to maintaining and updating information in WM (Morton & Munakata, 2002). Put another way, successful switchers use WM and CF to keep and update abstract active rule representations, while “perseverators” get stuck in stimulus-specific latent representations. We have shown in previous studies that high touchscreen users are faster and find more often a salient perceptual stimulus (Portugal, Bedford, Cheung, Gliga, & Smith, 2021; Portugal, Bedford, Cheung, Mason, & Smith, 2021). We think this can be indicative of greater learning of the regularities of stimuli-reward on-screen, and we now hypothesize that high users might have stronger representation of the pairing between perceptual stimuli and response, which could lead to failures in shifting task rule (e.g. the pre-switch stimuli-response representation is stronger), and in updating the task rule (e.g. in the delayed alternation task the pairing between the last successful door side and reward is stronger). Future studies should investigate whether faster bottom-up attention (indexing stronger stimuli-reward associations) might be related to deficits in CF and WM.

Another related developmental theory of EF that has addressed the DCCS difficulties focused on attentional inertia in, or poor disengagement from, the pre-switch stimuli-response pairing (Kirkham et al., 2003). We have shown in a previous study that long-term high users of touchscreens struggle with attention disengagement (Portugal, Bedford, Cheung, Mason, & Smith, 2021), so future studies should also investigate whether attention disengagement abilities might relate to CF and WM performance.

Another explanation for children's working-memory/cognitive flexibility difficulties could be due to their limited cognitive capacity. To explain the short-term effects of television in early childhood, it has been proposed that television viewing depletes cognitive resources available for EF right after viewing (Lillard, Li, & Boguszewski, 2015). If this is the case for touchscreen use as well, then high users may frequently experience cognitive resource depletion, and thus struggle with working-memory/cognitive flexibility. However, according to this theory, these limited resources would also compromise performance on impulse/self-control, but high and low users of touchscreens in our study did not differ in this component (although note the significant negative association between IC and viewing of adult-directed TV). In this regard, it could be that the IC tasks in our battery were less challenging for our age range, thus not sensitive enough to capture an effect of touchscreen use on IC – see the case of the Glitter Wand task in which only 7 in 46 children did not wait for the release of the prohibition. In light of the popular concerns about the digital media effects on the developing mind, it would be interesting in future studies, to dissociate the associations of IC with the early childhood media environment, including how different platforms and types of use/content of programming might contribute to its development.

It is important to note that the association between touchscreen user group and WM/CF no longer reached significance after controlling for background TV viewing, although the effect remained in the same direction. Given the small sample size, this may be due to a lack of statistical power, and results should be replicated in future studies. It may also be that the effect of touchscreen use on WM/CF is not specific to touchscreen devices but rather it is related to the child's broader media environment. Since our measure of background TV does not objectively measure children's TV viewing, and exposure via background TV will depend on factors such as number of TVs and how many hours children spend at home (i.e. whether they are in nursery, what time they go to bed), there is a need for more nuanced, objective methods in future studies to accurately measure duration, content and context of young children's media use.

The present study is also unable to test the causal direction of the association between touchscreen use and CF/WM. An alternative explanation for these findings is that children who struggle with working-memory/cognitive flexibility are more predisposed to request and engage with touchscreen technologies, because it provides them with a more regular environment and perceptual stimuli-response representations, or they are more likely to be given a touchscreen as a tool to support their EF difficulties, e.g., as a pacifier. However, our study benefited from a longitudinal design where cognitive and temperamental traits (which could be indicative of developmental differences) were assessed prior to the time point investigated here, which argues against the hypothesis that these results are driven by pre-existing differences (related to EF) between user groups.

This study also investigated how EF was organised at 3.5 years. Contrary to a tri-dimensional view of EF, where CF, WM, and IC are dissociated from each other, the correlations among measures in our sample showed a dissociation between measures tapping WM and CF, and measures tapping IC or impulse/self-control. This is in line with recent evidence that during toddlerhood and pre-school WM and CF development interacts substantially (Hendry et al., 2016; Hendry & Holmboe, 2021; Scionti & Marzocchi, 2021): abilities related to maintaining, updating, and shifting task set (an abstract representation of a goal or rule) in increasing levels of complexity. Further, impulse/self-control measures related to cool IC (measured by pre-potent motor response inhibition tasks) were not associated with *hot* IC measures related to delay of gratification, which has also been evident in previous studies (e.g.Carlson & Moses, 2001; Murray & Kochanska, 2002).

The design of this study has many strengths. First, it uses multiple measures of traditional and novel, age-appropriate EF tasks across a range of contexts, and combines a data- and theory-driven approach to study how EF is profiled in the sample. Second, the longitudinal design, with matched temperament scores at 12 months, allows pre-exisiting individual differences in EF precursors to be controlled for. A clear limitation of the current study is the modest sample size, which precluded a factor-analysis approach to test for longitudinal associations with EF compositie measures or its associations with attention measures. Despite this limitation, the descriptive statistics and the group comparisons for the measures of each individual experimental task tend to support the results from the composite analysis, i.e. for the WM EF lab-tasks (Delayed Alternation and Spin the pots) the high users had a consistent reduced mean score, and the difference between the groups for the DCCS (CF measure) was significant. Further, our sample has relatively high SES (high maternal education) and no atypicalities in development – while this made it possible to match user groups, it might also have lessened negative (e.g. low SES families might not have access to paid educational content) and positive (e.g. parents of children with neurodevelopmental disorders might benefit from content developed specifically for their children) effects of touchscreen use on executive functions. Our parent-report measure of touchscreen use is a further limitation, which resulted in our use of a binary grouping for touchscreen use, to minimise the effects of reporter bias. While dichotomising variables can reduce power, increase the risk of false positives and mask non-linear associations (Altman & Royston, 2006), correlational results remained substantively similar.

## 5 Conclusion

In this study, we investigated EF performance in preschool children with high and low touchscreen use, finding evidence that, as a group, high users show reduced EF performance on measures related to working-memory and cognitive flexibility, although no effects were found for impulse/self control. While this is an observational study, causality cannot be inferred, HU and LU were matched on a parent-report Effortful Control measure at 12 months, reducing the likelihood that high touchscreen use is a consequence of pre-exisiting individual differences in EF precursors. To further our understanding of why high users might fail to effortfully switch and maintain goals and task set representations, it is important that future studies investigate the associations of EF performance with abilities related to attention control, and dissociate between different types and contexts of touchscreen use.

## Credit author statement

Ana Maria Portugal: Conceptualization; Data curation; Formal analysis; Investigation; Methodology; Project administration; Resources; Software; Visualization; Roles/Writing - original draft; Writing - review & editing. Alexandra Hendry: Conceptualization; Methodology; Resources; Software; Writing - review & editing. Tim J. Smith: Conceptualization; Data curation; Funding acquisition; Methodology; Project administration; Supervision; Roles/Writing - original draft; Writing - review & editing. Rachael Bedford: Conceptualization; Data curation; Formal analysis; Funding acquisition; Methodology; Supervision; Validation; Roles/Writing - original draft; Writing - review & editing.

## Funding statement

The TABLET project was funded by a Philip Leverhulme Prize (PLP-2013–028) to TS. AMP was funded by an ESRC studentship (1,629,935) and a Welcome Trust/Birkbeck ISSF Postdoctoral Fellowship. AH is supported by the Scott Family Foundation Junior Research Fellowship in Autism, University College, University of Oxford and an NIHR and Castang Foundation Advanced Fellowship (NIHR300880). TS and RB were funded by the Nuffield Foundation (FR-000022056). RB was funded by an Henry Wellcome Postdoctoral Fellowship and King's Prize Fellowship (204823/Z/16/Z). For the purpose of open access, the author has applied a Creative Commons Attribution (CC-BY) licence [where permitted by UKRI, ‘Open Government Licence’ or ‘Creative Commons Attribution No-derivatives (CC-BY-ND) licence may be stated instead] to any Author Accepted Manuscript version arising.

## Ethics approval statement

The study was approved by the Birkbeck Psychological Sciences ethics board (Ref 141570 and 171821) which operate according to the British Psychological Sciences, ESRC ethical guidelines and adhere to the Declaration of Helsinki. Parents provided written informed consent on behalf of their children.

## Conflict of interest disclosure

The authors declare no competing interests.

## Data Availability

The datasets generated during and/or analysed during the current study can be accessed in OSF at https://osf.io/kavbd/?view_only=a132298b398344d881505ed0d665c507.
