# Supplementary material for: Do pre-schoolers with high touchscreen use show executive function differences?
Source: Comput Human Behav. 2023 Feb;139:107553. doi: 10.1016/j.chb.2022.107553 (PMC9746296; doi:10.1016/j.chb.2022.107553)
Supplement: Multimedia component 1 [file mmc1.docx]

**Appendix 1: pre-registered multivariate comparisons analysis at 3.5 years**

The analysis plan^[[1]](#footnote-1)^ for the 3.5-year visit was pre-registered prior to data processing and included hypotheses and plans related to various studies (including some already published, references removed for blind review). With regards to the current study, a plan was initially made to measure EF in 3 sub-components – working-memory (WM), inhibitory control (IC), and cognitive flexibility (CF) – and test performance between high and low users of touchscreen devices separately for screen-based tasks and real-world tasks (H2 a-c). While integrated-EF (H2d) and parent-report measures of IC (H2c) were also proposed to be tested in the pre-registration, these investigations were not reported in the current paper as we believe they should be reported as separate studies.

For the screen-based tasks, differences were planned to be tested using a MANOVA with the Delayed Alternation Score (WM), the Go/No-Go d’ statistic (cool-IC) and the Switch task conflict accuracy (CF) as dependent variables, and usage group (high, low) as fixed factor. The latter task, the Switch task, was completed but not analysed in the main study. This task was designed to assess CF, requiring children to pick and tap a door in the screen where a character was hidden, with changing rules for the pairing character and house. However, this task had not been validated in the age range of our study at the time of pre-registration, and performance, indexed by accuracy, tended to be better post-switch (Mean = .92, testing block) than pre-switch (Mean = .88, practice block, paired t-test *p* = .08), suggesting this task did not measure CF in our sample. Further, performance on this task, including the pre-registered accuracy in conflict trials, was not related to performance on the DCCS (and was related to measures of IC), which supported the decision to exclude this task from the main analysis. However, we report here the results.

For the real-world tasks, differences were planned to be examined using a MANOVA with the Spin the pots score (WM), the Snack delay score (hot-IC) and the DCCS ability to switch (CF) as dependent variables, and usage group (high, low) as fixed factor. The Glitter Wand was not mentioned in the plan, but was added later to the main study as it was found to be significantly related to Snack delay and it is a validated measure of Impulse/Self-control.

Table 1. Correlations between observed measures, represented as Spearman’s rho rs (p-value).

| Measure (Max N) | 2. | 3. | 4. | 5. | 6. | 7. Glitter Wand Latency |  |
| --- | --- | --- | --- | --- | --- | --- | --- |
| Screen-based tasks | | | | | | | |
| 1. Delayed Alternation Score (46) | .20 (.21) | .16 (.35) | **.34* (0.02)** | **.28’ (.06)** | .10 (.52) | .06 (.72) |  |
| 2. Switch Task, accuracy in conflict trials (42) |  | **.40* (.02)** | .08 (.62) | .18 (.26) | **.32* (.04)** | **.26’ (.09)** |  |
| 3. Go/No-Go D’ (38) |  |  | 0.07 (0.66) | 0.04 (0.84) | 0.14 (0.41) | 0.18 (0.27) |  |
| Table-top tasks | | | | | | | |
| 4. Spin the pots Score (46) |  |  |  | 0.24 (0.11) | -0.15 (0.32) | -0.05 (0.76) |  |
| 5. DCCS Correct sorted (46) |  |  |  |  | -0.16 (0.29) | -0.11 (0.48) |  |
| 6. Snack Delay Score (46) |  |  |  |  |  | **0.40** (0.01)** |  |

* p-value < 0.05, ‘ p-value < 0.1

Two MANOVA models were run with touchscreen usage group as a predictive factor of either EF on touchscreen-based tasks or on table-top tasks. The multivariate model predicting EF on touchscreen-based tasks was not significant, F (3, 31) = 1.400, *p* = 0.261, η^2^ = 0.119; neither was the multivariate model predicting EF on table-top tasks, F (3, 42) = 1.303, *p* = 0.286, η^2^ = 0.085.

In addition, touchscreen usage group comparisons on all the EF tasks were tested using a univariate non-parametric test (Mann-Whitney), given that all scores were non-normally distributed (Shapiro-Wilk test p-value < 0.01), and reported in Table 1.

Table 2. Performance statistics and multiple comparison analysis on the EF measures, by touchscreen use group at 3.5 years. All variables index performance on EF in a way that a higher value is better EF.

|  | LUs | HUs | Test statistic | Uncorrected p-value |
| --- | --- | --- | --- | --- |
| Touchscreen-based Tasks | | | | |
| Delayed Alternation WM Score  M(SD)  n (girls) | 6.84 (5.18)  19 (12) | 4.63 (3.65)  27 (11) | U = 202,  z = -1.224 | 0.221 |
| Switch Accuracy in conflict  M(SD)  n (girls) | 0.93 (0.09)  17 (10) | 0.90 (0.10)  25 (9) | U = 159.5,  z = -1.391 | 0.164 |
| Go/No-Go D’:  M (SD)  n (girls) | 2.40 (0.78)  16 (9) | 2.31 (0.80)  22 (9) | U = 170,  z = -0.178 | 0.859 |
| Table-top Tasks | | | | |
| Spin the pots Score  M (SD)  n (girls) | 12.32 (3.13)  19 (12) | 11.59 (3.38)  27 (11) | U = 217.5,  z = -0.877 | 0.381 |
| DCCS # Cards sorted  M (SD)  n (girls) | 3.53 (2.99)  19 (12) | 1.89 (2.69)  27 (11) | U = 178,  z = -1.960 | **0.050** |
| Snack Delay Sum Score  M (SD)  n (girls) | 21.05 (3.36)  19 (12) | 21.56 (2.34)  27 (11) | U = 271.5,  z = 0.339 | 0.734 |
| Not pre-registered measures | | | | |
| Glitter Wand Latency  M (SD)  n (girls) | 31.89 (8.81)  19 (12) | 32.59 (7.39)  27 (11) | U = 260,  z = 0.125 | 0.901 |

Descriptively, high users had a consistently poorer performance on all EF tasks, except for the Delayed Gratification ones (*hot* IC). The difference between groups performance was significant on the DCCS (which indexes CF).

**Appendix 2: summary of longitudinal analysis**

Table 3. Summary of univariate ANOVA results, testing the effects of concurrent use (3.5-year use, coded dichotomously as greater than or equal to 15 min/day), past use (12-month use, coded dichotomously as greater than or equal to 10 min/day), and longitudinal use (i.e., stable touchscreen use, coded based on consistent use from 12-18 months to 3.5 years).

|  | ANOVA  (high, low at 3.5 years) | ANOVA  (high, low at 12 months) | ANOVA  (high, low longitudinal) |
| --- | --- | --- | --- |
| Working-Memory/Cognitive Flexibility  Univariate ANOVA  n (girls) | F(1,44)=4.498, ***p*=0.040**  46 (23) | F(1,44)=0.627, *p*=0.433  46 (23) | F(1,32)=2.334, *p*=0.136  34 (16) |
| Impulse/Self-Control  Univariate ANOVA  n (girls) | F(1,44)=0.074, *p*=0.787  46 (23) | F(1,44)=1.556, *p*=0.219  46 (23) | F(1,32)=0.059, *p*=0.810  34 (16) |

1. Removed for blind review [↑](#footnote-ref-1)
